# 

**Published:** 1866-01

**Authors:** 


### CONTENTS.

ORIGINAL ESSAYS AND COMMUNICATIONS.

Dental Materia Medica.............................. 9

Address.............................. 14

The Province and Duties of the Dentist.............................. 19

Address.............................. 25

PROCEEDINGS OF SOCIETIES.

Central Ohio Dental Association.............................. 32

SELECTIONS.

Double Fracture of the Inferior Maxilla.............................. 38
Anæsthetics.............................. 40
The Preparation of Pure Chloroform.............................. 41
Necrosis.............................. 42
Nitrous Oxide as an Anæsthetic.............................. 44
Phenic Acid as an Antiseptic.............................. 45
A New Blow-Pipe.............................. 45
A Simple, Safe and Easy Mode of Preparing Oxygen.............................. 46
Necrosis of the Jaw from Decayed Tooth.............................. 46
Obituary of Dr. W. R. Hodgen.............................. 47
Order of Business.............................. 48
Michigan Dental Association.............................. 48

EDITORIAL.

The New Year.............................. 49
Professional Liberty and Restraint.............................. 50
Central Ohio Dental Association.............................. 52
Mississippi Valley Dental Association.............................. 53
White's "Universal Porte Polisher.".............................. 53
The Ohio Dental College Association.............................. 53
Bibliographical.............................. 54

